# The Effect of Dimethyl Sulfoxide on the Lysozyme Unfolding Kinetics, Thermodynamics, and Mechanism

**DOI:** 10.3390/biom9100547

**Published:** 2019-09-29

**Authors:** Timur Magsumov, Alisa Fatkhutdinova, Timur Mukhametzyanov, Igor Sedov

**Affiliations:** Chemical Institute, Kremlevskaya 18, Kazan Federal University, 420008 Kazan, Russia

**Keywords:** protein denaturation, protein unfolding, lysozyme, unfolding kinetics, differential scanning calorimetry, circular dichroism

## Abstract

The thermal stability of proteins in the presence of organic solvents and the search for ways to increase this stability are important topics in industrial biocatalysis and protein engineering. The denaturation of hen egg-white lysozyme in mixtures of water with dimethyl sulfoxide (DMSO) with a broad range of compositions was studied using a combination of differential scanning calorimetry (DSC), circular dichroism (CD), and spectrofluorimetry techniques. In this study, for the first time, the kinetics of unfolding of lysozyme in DMSO–water mixtures was characterized. In the presence of DMSO, a sharp decrease in near-UV CD and an increase in the fluorescence signal were observed at lower temperatures than the DSC denaturation peak. It was found that differences in the temperatures of the CD and DSC signal changes increase as the content of DMSO increases. Changes in CD and fluorescence are triggered by a break of the tertiary contacts, leading to an intermediate state, while the DSC peak corresponds to a subsequent complete loss of the native structure. In this way, the commonly used two-state model was proven to be unsuitable to describe the unfolding of lysozyme in the presence of DMSO. In kinetic studies, it was found that even high concentrations of DMSO do not drastically change the activation energy of the initial stage of unfolding associated with a disruption of the tertiary structure, while the enthalpy of denaturation shows a significant dependence on DMSO content. This observation suggests that the structure of the transition state upon unfolding remains similar to the structure of the native state.

## 1. Introduction

The use of mixed aqueous–organic solvents allows enzyme-catalyzed reactions to be conducted with substrates that are poorly soluble in water, to change the selectivity of these reactions, and sometimes to make them proceed in the reverse direction [1,2,3]. However, with few exceptions [4,5], the addition of organic cosolvents leads to a decrease in the stability of the native structure of the enzyme proteins [6], a decrease in the denaturation temperature [7,8,9,10], and a reduction of the catalytic activity [11,12,13].

Besides the lower thermodynamic stability of proteins in aqueous–organic mixtures reflected in lower denaturation temperatures, the kinetic stability of the native state and the thermodynamic functions of activation of the unfolding process are also affected by the presence of organic cosolvents. Moreover, organic denaturants may influence the mechanism of unfolding and the structure of the transition state. While there are many papers dedicated to the effects of organic cosolvents on the thermodynamic stability of proteins [9,14,15,16,17,18], the kinetics and mechanisms of protein unfolding in binary aqueous–organic solvents remain poorly explored.

Hen egg-white lysozyme is often used as an object to study the influence of various factors on the thermal stability, structure, and activity of enzyme proteins. The dependence of its denaturation temperature on the pH [19], ionic strength [20], pressure [21], and concentration of various organic cosolvents and denaturants [8,22,23,24] has been explored. In particular, several works [14,25,26,27,28] have focused on the behavior of lysozyme in the presence of dimethyl sulfoxide (DMSO), a low toxic organic solvent. DMSO and its mixtures with water are used in the solubilization of drugs (e.g., Onyx^®^, ethylene vinyl alcohol copolymer dissolved in DMSO [29]), as penetrating agents and cryoprotectants for cells and tissues, and as local analgesic and anti-inflammatory agents (e.g., Dolobene^®^ [30], Rimso-50^®^, an aqueous solution containing 50 weight% (20 mole%) of DMSO [31]).

The dependence of the temperature and enthalpy of lysozyme denaturation on the concentration of DMSO was studied in [25]. It was found that in the mixtures containing about 40 mole% of DMSO, lysozyme denatures at room temperature. Using dynamic light scattering, small-angle neutron scattering, infrared and circular dichroism spectroscopy [26], and ^1^H NMR spectroscopy [27], the chemical denaturation of lysozyme at room temperature under the influence of increasing concentrations of DMSO was shown to first lead to a loss of the tertiary structure while preserving part of the native secondary structure, which disrupts at higher concentrations of DMSO.

The kinetic parameters of the lysozyme unfolding during its denaturation in the presence of DMSO have never been determined. Interestingly, some other denaturants were shown to accelerate the process of protein unfolding, but not to significantly change the value of its activation energy or enthalpy. Segawa and Sugihara [32] found that the addition of small quantities of guanidinium chloride (up to 3.2 M), isopropanol (up to 3.9 M, or 10 mole%), or n-propanol (1.6 M) speed up the unfolding of lysozyme, but the enthalpy of activation of this process remains almost the same regardless of the nature and concentration of the considered denaturants. They concluded that the conformation of lysozyme in the transition state of the unfolding process remains compact and similar to its native state, while the enthalpy of activation of unfolding can be attributed to a disruption of the long-range interactions between the structural domains. For another protein, trypsin, the addition of moderate amounts (17.5%) of ethanol was shown not to change the activation energy of unfolding [33]. It is unknown how the activation energy changes at higher concentrations of the organic cosolvents.

In the present work, we study the influence of relatively high concentrations of DMSO on the rate and activation energy of lysozyme unfolding. The obtained data, together with the thermodynamic parameters of denaturation in DMSO-water mixtures, will be used to deepen our understanding of the mechanisms of this process in the presence of different concentrations of DMSO.

## 2. Materials and Methods

### 2.1. Materials

The hen egg-white lysozyme (HEWL, Sigma Aldrich 62970, St. Louis, MO, USA) was used without preliminary purification. DMSO (Komponent-Reaktiv, Moscow, Russia, 99%) was additionally purified using a standard method [34], including drying with calcium hydride and distillation in vacuo. Deionized water without a buffer was used in all experiments in order to avoid unwanted effects when mixed with DMSO, such as precipitation of the buffer components or demixing. Protein solutions were prepared by dissolving a weighed sample of lysozyme in the necessary amount of pure water or preliminarily prepared mixture of water and DMSO with a certain composition. The concentration of the aqueous solution calculated from weighing data was confirmed by measuring the optical density at a 280 nm wavelength (the extinction coefficient of lysozyme equals 2.65 mg·mL^−1^·cm^−1^ [35]).

### 2.2. The Differential Scanning Calorimetry (DSC) Method

The temperatures and enthalpies of denaturation of lysozyme at various concentrations of DMSO were determined using a NanoDSC capillary differential scanning calorimeter (TA Instruments, New Castle, DE, USA). The calorimeter was calibrated according to the previously described procedure [36]. The temperature calibration was verified by measuring the temperature of the peak maximum for the solution of lysozyme in the 0.1 M glycine-HCl buffer with pH = 2.4. The protein solution in a 300 μL capillary cell of the calorimeter was heated from 283 to 373 K at a constant rate from 0.1 to 2 K·min^−1^. The lysozyme concentration was 5 mg·mL^−1^. The reference cell was filled with a protein-free solvent containing the same concentration of DMSO. For all studied lysozyme solutions, the decrease in concentration from 5 down to 0.5 mg·mL^−1^ at the same scan rate does not change the position of the peak maximum. Thus, it is possible to compare calorimetric data with the results of spectroscopic studies at lower concentrations of lysozyme. This also suggests that the observed DSC peak corresponds only to the protein unfolding process without a significant contribution of aggregation. The literature data confirm that, at the used concentrations of lysozyme, no immediate aggregation of the denatured protein occurred in pure water [37], as well as in its mixtures with DMSO [26].

### 2.3. Circular Dichroism (CD) Spectroscopy Study of Unfolding Equilibrium

The temperature-induced changes in the intensity of the CD signal of lysozyme solution in water or DMSO-water mixtures were studied using a Jasco J-1500 spectropolarimeter in a quartz cuvette with a 10 mm optical path length. During the experiment, the cuvette was heated from 283 to 358 K, with a heating rate of 0.2 K·min^−1^. The temperature was controlled using a thermocouple. To study the changes in the tertiary structure of the protein, the signal intensity at a 290 nm wavelength was monitored. The protein concentration was 0.5 mg·mL^−1^. For a solution in pure water, an additional experiment was carried out to obtain information on the changes in the secondary structure by following the CD signal at a 222 nm wavelength. In this case, a 0.1 mg·mL^−1^ lysozyme solution was used. Strong absorption of DMSO in the far UV range prevents similar experiments from being performed in its mixtures with water.

### 2.4. Spectrofluorimetric Study of Unfolding Equilibrium

The intensity of the lysozyme fluorescence at different temperatures after several minutes of equilibration was measured using a Cary Eclipse fluorescence spectrophotometer (Agilent). The excitation wavelength was 280 nm, and the emission intensity was measured in the range of 320–380 nm.

### 2.5. Spectrofluorimetric Study of Unfolding Kinetics

The kinetics of lysozyme unfolding was studied using a rapid mixing accessory SFA-20 (Hi-Tech Scientific, Bradford-on-Avon, UK) and a Cary Eclipse fluorescence spectrophotometer. The aqueous solution of the protein and the aqueous solution of DMSO were mixed at a 1:20 volume ratio at different temperatures close to the unfolding temperature determined from CD studies. The concentration of lysozyme in the initial aqueous solution was 0.21 mg·mL^−1^, and in solutions after mixing −0.01 mg·mL^−1^ (7 × 10^−7^ M). The concentrations of DMSO solutions were chosen to achieve the mole fractions of DMSO *x*_1_ in solutions after mixing equaling 0.25, 0.3, or 0.35. Changes in the fluorescence intensity were followed at an excitation wavelength of 280 nm and an emission wavelength of 345 nm.

It is necessary to minimize the changes in the temperature of the solution inside the cell after mixing. Following from the data on the excess enthalpies of mixing [38], the addition of water to the mixtures containing DMSO at a 1:20 volume ratio at adiabatic conditions should not lead to a rise in temperature of more than 2 K. In our experiments, the cuvette is thoroughly thermostated. In addition, the first 5 s were cut from each kinetic curve, which should be sufficient for temperature equilibration.

## 3. Results

### 3.1. DSC Experiments

**Figure 1:**
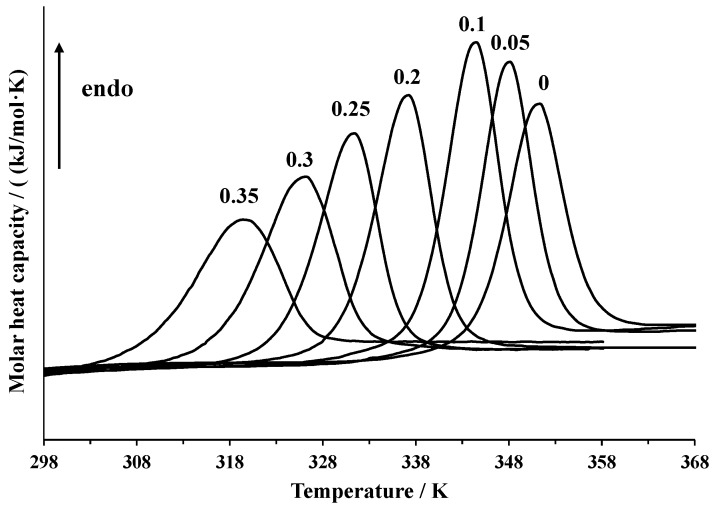
DSC curves of denaturation of lysozyme in DMSO-water mixtures and pure water at a 1 K·min^−1^ heating rate.

Differential scanning calorimetry was used to assess the thermal stability of lysozyme at different concentrations of DMSO and analyze the scan rate dependence of the position of the calorimetric peak. The raw data are shown in Figure 1. In Table 1, the temperatures of the peak maximum and enthalpies of denaturation of lysozyme in the DMSO-water mixtures and pure water at 1 K·min^−1^ heating rate obtained by peak integration are given. The calorimetric enthalpy of denaturation *Δ_d_H_cal_* = 494 ± 10 kJ·mol^−1^ in pure water agrees with the previously published results of other authors. In the literature, the values of *Δ_d_H_cal_* = 536 kJ·mol^−1^ (pure water, 6 times recrystallized lysozyme) [25], 454 kJ·mol^−1^ (pure water, lysozyme lyophilized at pH = 8) [4], 409 kJ·mol^−1^ (0.010 M phosphate buffer, pH = 6.89) [39], 481 kJ·mol^−1^ (0.020 M glycine buffer, pH = 2.5) [40], 476 kJ·mol^−1^ (pH = 2.5) [41], and 464 kJ·mol^−1^ (0.050 M phosphate buffer, pH = 6) [19]) were reported.

Since DMSO acts as a denaturating agent, an increase of its concentration in the mixture leads to a shift in the peak towards lower temperatures. The addition of small quantities of DMSO (up to *x*_1_ = 0.1) increases the denaturation enthalpy, while further growth of DMSO content in the mixture leads to a decrease. This concentration dependence of denaturation enthalpy was previously explained by the preferential solvation of the native state of lysozyme by the DMSO molecules and the denatured state by water molecules [25]. However, other experimental [42] and simulation [43] studies show that preferential solvation with DMSO increases upon denaturation. An alternative explanation involves a hypothesis of some structural changes of the protein in DMSO-water mixtures [14]. The enthalpies obtained in the present work are close to those reported in [28] and are somewhat lower than those reported by Kamiyama et al. [25]. We have also recorded the DSC curves in mixtures with a higher content of DMSO (*x*_1_ = 0.35) than in any of the preceding works.

In Table 2, the temperatures of the peak maxima at different heating rates are compared. The increase of the heating rate from 0.5 to 2 K·min^−1^ results in a peak maximum shift of approximately 1 K in pure water and about 3 K in the mixture containing 30 mole% of DMSO. These data allow one to estimate the equilibrium denaturation temperatures *T_d(eq)_* by extrapolating to zero scanning rate, which are given in the last column of Table 2. The enthalpies of denaturation in the same solvent mixture at different heating rates coincide with each other.

In the cooling curves recorded immediately after heating, the renaturation peaks appear in experiments with any DMSO concentration. The maxima are observed at temperatures slightly lower than the equilibrium denaturation temperature. In general, denaturation is partially reversible. If the cooling starts immediately after reaching the endset temperature of the denaturation peak, then the value of the enthalpy of denaturation obtained from the subsequent heating scan will be close to that from the first scan. However, prolonged exposure of lysozyme to temperatures above *T_d_* leads to irreversible chemical degradation processes, resulting in a decrease in the degree of renaturation upon cooling. These processes include oxidation and solvolysis of the side chains, as well as other reactions [44,45].

### 3.2. Circular Dichroism Spectroscopy

**Figure 2:**
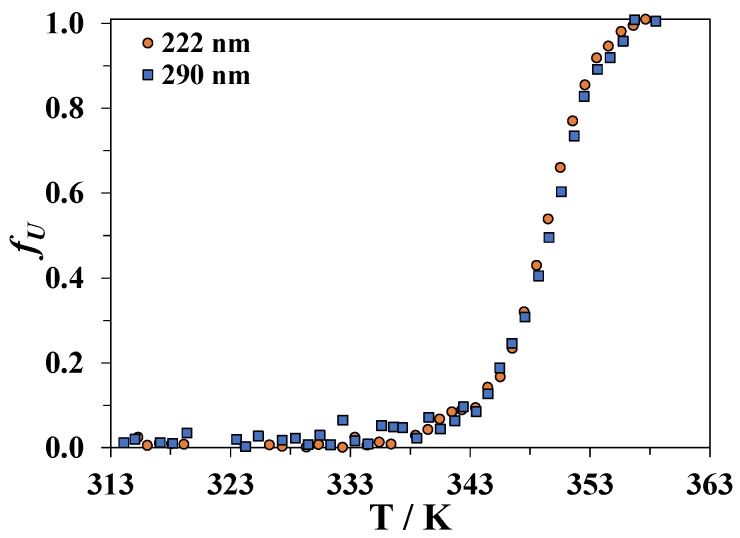
Dependence of the fraction *f_U_* of the lost secondary (CD signal measured at 222 nm) and tertiary (CD signal measured at 290 nm) structure of lysozyme in an aqueous solution as a function of temperature.

A comparison of the changes in the secondary and tertiary structure of lysozyme upon heating its aqueous solution is shown in Figure 2. The fraction of the protein molecules that have lost their native (tertiary or secondary) structure is denoted as *f_U_* and was calculated from the CD data using the following formula:

(1) $$f_{U}=\frac{\theta -(\theta_{N}+m_{N}T)}{\left(\theta_{U}+m_{U}T)-(\theta_{N}+m_{N}T\right)},$$

where θ is the measured CD signal at a chosen wavelength, $\theta_{N}+m_{N}T$, and $\theta_{U}+m_{U}T$ are the linear approximations of the baselines for the native and unfolded forms of the protein, respectively.

Figure 2 indicates that the tertiary and secondary structures of lysozyme heated in water are simultaneously disrupted. This result confirms the previously reported data [46].

Due to the strong absorption of DMSO at wavelengths below 270 nm, CD measurements cannot be used to study the changes of the secondary structure of proteins in DMSO-water mixtures. Thus, we studied only the changes of the tertiary structure. At 293 K, lysozyme has a similar near-UV CD spectra in the mixtures containing less than 37.5 mole% (70 volume%) DMSO [26], which indicates a similar structure of the native state in all the studied mixtures. The unfolded form has a low intensive and uninformative CD signal that does not allow one to draw conclusions about the difference in its structure in different mixtures.

**Figure 3:**
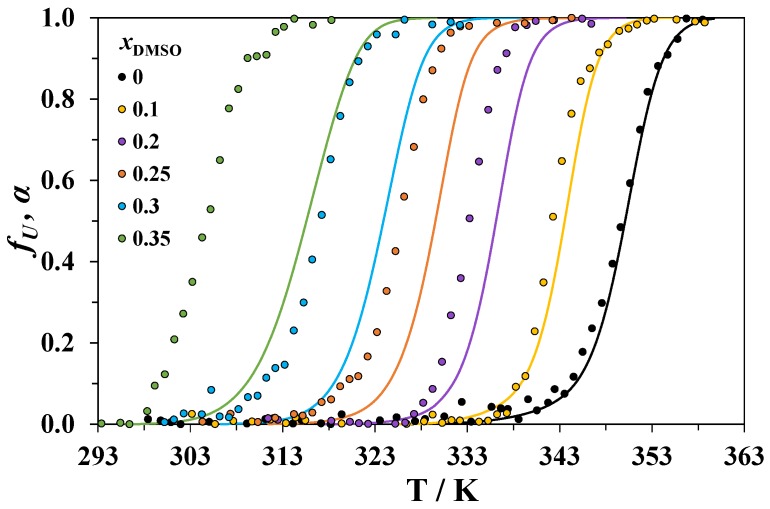
Temperature dependencies of the degree of conversion of lysozyme from the DSC data at a 0.5 K·min^−1^ heating rate (*α*, solid lines) and a fraction of the lost tertiary structure from the CD measurements (*f_U_*, circles). The data for the mixtures with different mole fractions of DMSO *x*_1_ are shown in different colors.

The temperature dependences of *f_U_* at 290 nm for lysozyme heated in solvent mixtures with different compositions is shown in Figure 3. The obtained curves can be compared with the temperature dependencies of the DSC-derived degree of protein conversion *α*, which were obtained by integration of the calorimetric curves recorded at the lowest heating rate (0.5 K·min^−1^). These dependencies are also shown in Figure 3. The raw plots of θ as a function of temperature are given in the Supplementary Material.

For all the studied systems, the differences between the temperature *T_d_* of the DSC peak maximum (at 0.5 K·min^−1^ heating rate) and the temperature *T_CD_* corresponding to the maximum of the derivative $\frac{df_{U}}{dT}$ are given in Table 3. For the denaturation of lysozyme in water, *T_CD_* approximately equals *T_d_*. With an increasing concentration of DMSO, *T_CD_* increasingly diverges from *T_d_*. We must note that the values of *T_d_* obtained at a 0.5 K·min^−1^ heating rate can be slightly higher than those obtained at 0.2 K·min^−1^ (the scanning rate in CD experiments), but the differences between them should be very small in comparison with the observed *T_d_* − *T*_CD_ differences (see also Table 2).

In DMSO-rich mixtures, only a small part of the total heat of denaturation is absorbed when the *T_CD_* is reached (Figure 3). This means that the processes causing the change in the CD signal at a 290 nm wavelength are accompanied by small heat effects. The changes in the CD spectra in the near-UV region are usually attributed to the perturbations of the tertiary structure of proteins. In the early stage of unfolding, cosolvent molecules can penetrate into particular regions of the native protein and disrupt the interactions between chromophores [47,48], which affect the CD signal. It is likely that the observed drop in ellipticity corresponds to the beginning of the loss of the lysozyme tertiary structure, while the DSC peaks correspond to a complete disruption of both the tertiary and secondary structures. The values of *T_d_* from our calorimetric experiments are close to the temperatures of unfolding of the secondary structure of lysozyme in DMSO-water mixtures obtained from an analysis of the FTIR spectra [49]. With an increasing concentration of DMSO, we can expect enhanced penetration of its molecules into the protein globule leading to the appearance of the CD peak well before the complete unfolding of lysozyme occurs.

Previously, the difference between *T_d_* from DSC studies and *T_CD_* from near-UV or far-UV CD experiments has been observed for the unfolding of β-lactoglobulin A in an acidic solution [50] and a few other systems [51]. A higher thermal stability than the stability of the tertiary structure was demonstrated for the secondary structure in the denaturation of lysozyme in glycerol [46,52], the denaturation of ribonuclease A in the presence of alkylurea [53], and in the mixture of water with methanol [54].

The change in the CD signal of the solutions upon cooling immediately after heating indicates renaturation of the tertiary structure of lysozyme at temperatures slightly below *T_CD_*.

### 3.3. Spectrofluorimetry

**Figure 4:**
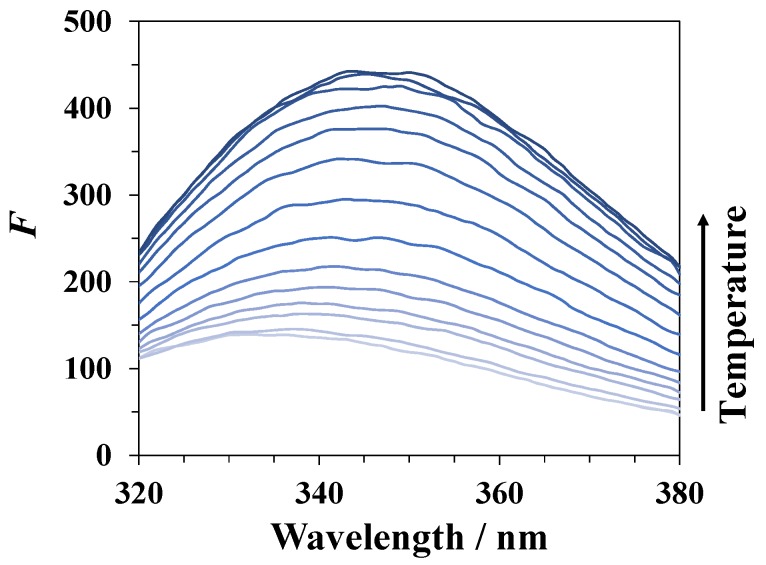
Changes in the emission spectrum of lysozyme with increasing temperature in a DMSO–water mixture with a DMSO mole fraction *x*_1_ = 0.30. The excitation wavelength is 280 nm.

**Figure 5:**
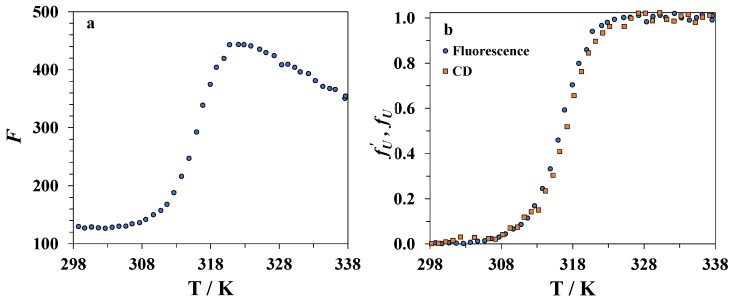
(**a**) Changes in lysozyme fluorescence intensity with an increasing temperature in the DMSO–water mixture with a DMSO mole fraction of *x*_1_ = 0.30. (**b**) The temperature dependence of the degree of unfolding of lysozyme as determined from the fluorescence (circles) and CD (squares) data in the DMSO–water mixture with a DMSO mole fraction of *x*_1_ = 0.30.

During denaturation of lysozyme in DMSO–water mixtures, an increase in its fluorescence intensity (see Figure 4) occurs due to a change in the environment of the fluorophore fragments in the protein molecule. The intensity of fluorescence begins to increase sharply when approaching the protein denaturation temperature, reaches a maximum value, and then decreases at higher temperatures, which is likely due to the temperature quenching of the fluorescence. An example of the temperature dependence of the fluorescence intensity for lysozyme in a mixture of water with 30 mole% of DMSO is shown in Figure 5a. We used the magnitude $f_{U}^{\prime }$ given by an equation similar to Equation (1) used in CD studies to characterize the degree of unfolding of the protein from the spectrofluorimetric data:

(2) $$f_{U}^{\prime }=\frac{F-(F_{N}+n_{N}T)}{\left(F_{U}+n_{U}T)-(F_{N}+n_{N}T\right)},$$

where *F* is the measured fluorescence intensity at a chosen wavelength, and $F_{N}+n_{N}T$ and $F_{U}+n_{U}T$ are the linear approximations of the baselines for native and unfolded forms of the protein, respectively. The temperature dependence of $f_{U}^{\prime }$ for lysozyme in the mixture containing 30 mole% of DMSO is shown in Figure 5b. It was found that the temperatures corresponding to the maximum of the derivative $\frac{df_{U}^{\prime }}{dT}$ are very close to the *T_CD_* values for the same mixture, i.e., the jump of the fluorescence intensity is linked with the beginning of the disruption of the tertiary structure of lysozyme. Thus, the kinetics of unfolding of the tertiary structure can be studied by following the time dependence of the fluorescence intensity of lysozyme at a constant temperature. The fluorescence signal is noise-free and stable compared to the CD signal, which allows one to conduct more accurate measurements, including evaluation of the activation energy from the temperature derivatives of the rate constants.

### 3.4. Notes on Kinetic Models of Denaturation

The possible existence of intermediates during denaturation is a key problem for the analysis of experimental data related to the kinetics of unfolding. In a number of works [32,51,52,55,56], the thermally induced unfolding of lysozyme in water was shown to be a single-step reversible process of the transition from a native (*N*) to a completely unfolded (denatured) (*U*) state in a homogeneous solution:

(3) $$N\overset{k_{u}}{\underset{k_{f}}{⇄}}U,$$

where *k_u_* and *k_f_* are the rate constants of unfolding and folding, respectively. The equilibrium constant for such a process is given by:

(4) $$K=\frac{\left[U\right]}{\left[N\right]}=\frac{k_{u}}{k_{f}}$$

A number of other works challenge this view, suggesting the existence of an intermediate state or states [40,57,58,59,60,61]. Our study of lysozyme denaturation in water and its mixtures with DMSO shows that, in pure water, both the secondary and tertiary structures are destroyed at the same time at a certain temperature, while in the mixtures containing DMSO, the tertiary contacts break before the complete loss of the native structure, and the differences in the temperatures of the CD and DSC peaks increase with an increasing concentration of DMSO. Thus, it is quite clear that we cannot consider denaturation of lysozyme in the presence of DMSO as a single-step process. At least two stages of unfolding must be distinguished. The second stage accompanied by the complete disruption of the native structure is notably slower and occurs at higher temperatures. However, the obtained data cannot rule out the possibility of a more complicated mechanism with multiple stages.

It is likely that even in pure water, unfolding of lysozyme cannot be considered as a single-step process. One argument in favor of a multistep mechanism is based on the reported values of the activation energy of unfolding (259 kJ·mol^−1^ [62,63]). This is much lower than the enthalpy of lysozyme denaturation, so single-step reversible unfolding should have had a large negative activation energy in the reverse process, which is very improbable.

**Figure 6:**
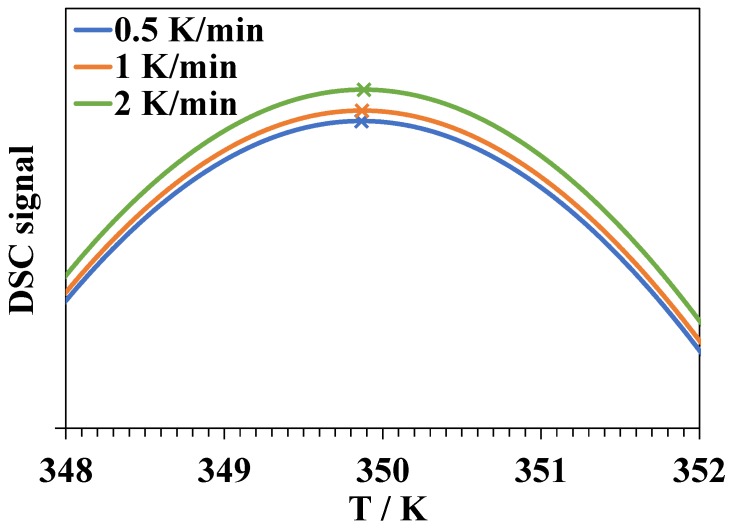
The results of the numerical simulation of the DSC curves at heating rates of 0.5, 1, and 2 K·min^−1^ for a hypothetical reversible single-step denaturation process in pure water.

Another argument is connected with the scan rate dependence of DSC peak position. It is obvious that in DSC experiments at sufficiently low heating rates *v*, the equilibrium of unfolding will be established at all the temperatures during the scan. The peak maximum at such scan rates is very close (but not exactly equal due to peak asymmetry) to the temperature at which K=1. By increasing the scan rate, the peak will shift towards higher temperatures since the equilibrium will no longer be established [64]. In Figure 6, we show the results of a numerical simulation of the DSC curves for a hypothetical reversible single-step denaturation process in pure water. This simulation was conducted using a program written by the authors. We assume that the lower limit of the rate constant of lysozyme unfolding in pure water at *T_d_* = 350 K equals 20 min^−1^ [32], the activation energy of unfolding is 259 kJ·mol^−1^, and the activation energy of folding is 56 kJ·mol^−1^ [65]. The shift of the peak maximum upon an increase of the heating rate from 0.5 to 2 K·min^−1^ did not exceed 0.02 K.

The experimentally observed peak shift of more than 1 K would have been observed if the rate constant of unfolding was significantly lower, or if the difference between the energies of activation of the forward and reverse processes was low, which indicates a small enthalpy of denaturation. In the case of a two or multi-step unfolding, the existence of a step satisfying one of these conditions may lead to the observed shift.

The possibility of the existence of a lysozyme unfolding intermediate was first discussed by Privalov [57]. In a later work [40], Privalov concluded that the shape of the DSC denaturation peak is better described with a three-state than with a two-state model of denaturation. A similar conclusion was recently developed by Mazurenko et al. [58]. In the work [59], the Raman spectra of the side groups of lysozyme were found to change at a lower temperature than the Amide I band of the protein backbone. This was attributed to the prior changes in the tertiary structure without unfolding the secondary structure. Simulations of unfolding of lysozyme in water at high temperatures [43,61] also show the existence of an intermediate state with partly disrupted native contacts that retains a compact structure. Our present findings provide more evidence for a non-single-step unfolding mechanism using different arguments.

### 3.5. Isothermal Kinetics of Lysozyme Unfolding

In accordance with the above analyses, a study of the time change in the fluorescence intensity of lysozyme in DMSO-water mixtures can provide information only about the kinetics of the initial stage of the unfolding process accompanied by the breaking of at least part of the tertiary contacts in the native structure of lysozyme. If we consider this stage as a reversible transition of the native state *N* into an unfolding intermediate *I* according to scheme $N\overset{k_{1}}{\underset{k_{-1}}{⇄}}I$, where both the forward and reverse processes are of the first order, and assume that each state independently contributes to the total fluorescence proportionally to its concentration, then the fluorescence intensity changes with time according to the following equation:

(5) $$F(t)=F(\infty )-\left(F(\infty )-F(0)\right)\exp\left(-\left(k_{1}+k_{-1}\right)t\right),$$

where F(0)*,* F(∞)*,* and F(t) are the fluorescence intensities at the initial moment of time, after prolonged equilibration, and at the moment of time *t*, respectively. Therefore, fitting the fluorescence intensity to the exponential dependence over time provides the sum of the rate constants $k_{1}$ of the forward step and $k_{-1}$ of the reverse step. The ratio $k_{1}/k_{-1}$ grows with temperature and should reach 1 at *T_CD_*. At higher temperatures, $k_{-1}$ becomes smaller than $k_{1}$. The apparent energy of activation is given by:

(6) $$E_{a}=-R\frac{d\ln(k_{1}+k_{-1})}{d(1/T)}=\frac{k_{1}E_{1}+k_{-1}E_{-1}}{k_{1}+k_{-1}}.$$

**Figure 7:**
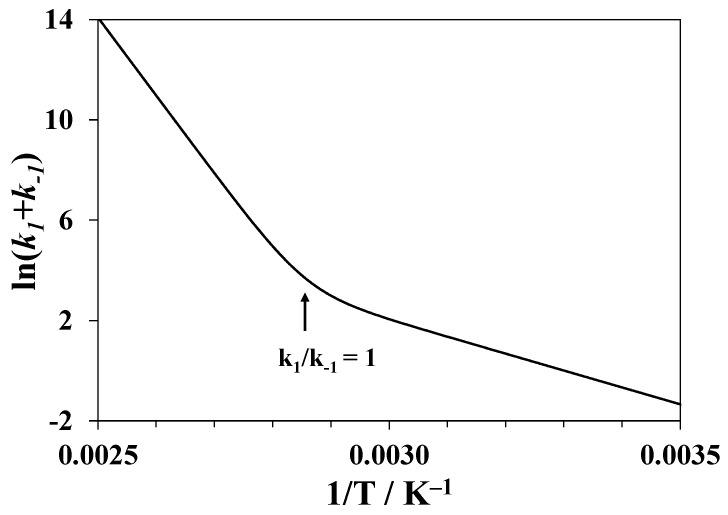
Results of numerical modeling for a single-step reversible process of unfolding of the tertiary structure of lysozyme in pure water: dependence of the logarithm of the sum of the rate constants of forward and reverse processes on 1/*T*.

This magnitude will change with an increasing temperature from the energy of activation of the reverse step $E_{-1}$ at $k_{1}/k_{-1}$ <<1 to the energy of activation of the forward step $E_{1}$ at $k_{1}/k_{-1}$ >> 1. An example is given in Figure 7 for the numerical modeling of the temperature dependence of $\left(k_{1}+k_{-1}\right)$ for unfolding of lysozyme in pure water using the rate constant values $k_{1}=k_{-1}$ = 20 min^−1^ at *T* = 350 K, the activation energies $E_{1}$ = 259 kJ·mol^−1^, and $E_{-1}$ = 56 kJ·mol^−1^. The bend point corresponds to 350 K.

Hence, in order to determine the activation energy of the tertiary structure unfolding $E_{1}$, one should consider only the values of $k_{1}+k_{-1}$ measured above *T_CD_*. Below *T_CD_*, the changes in the fluorescence are small since the degree of unfolding is also small. Therefore, the rate constants and the magnitude of $E_{-1}$ cannot be precisely determined using this method.

**Figure 8:**
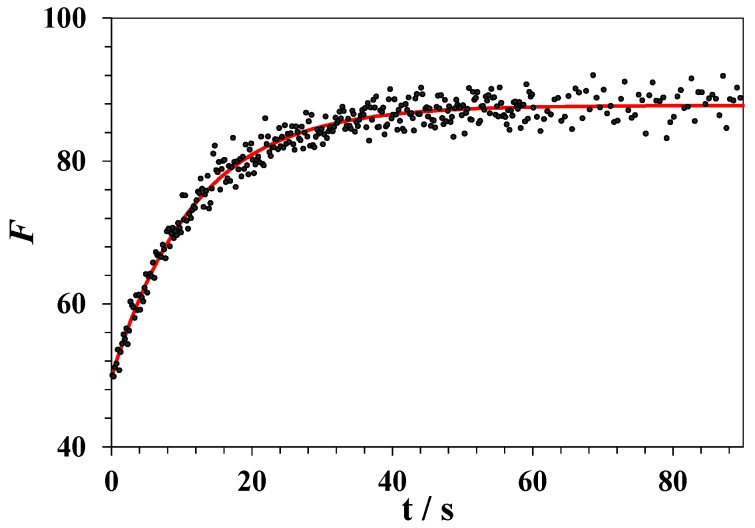
Changes in the fluorescence intensity upon unfolding of lysozyme in the DMSO–water mixture with a DMSO mole fraction of *x*_1_ = 0.25 at a constant temperature 327 K. The red line corresponds to an exponential approximation via Equation (5).

Equation (5) provides a good fit for the experimental data. An example of the plot of lysozyme fluorescence intensity against time recorded at a constant temperature above *T_CD_* is shown in Figure 8.

**Figure 9:**
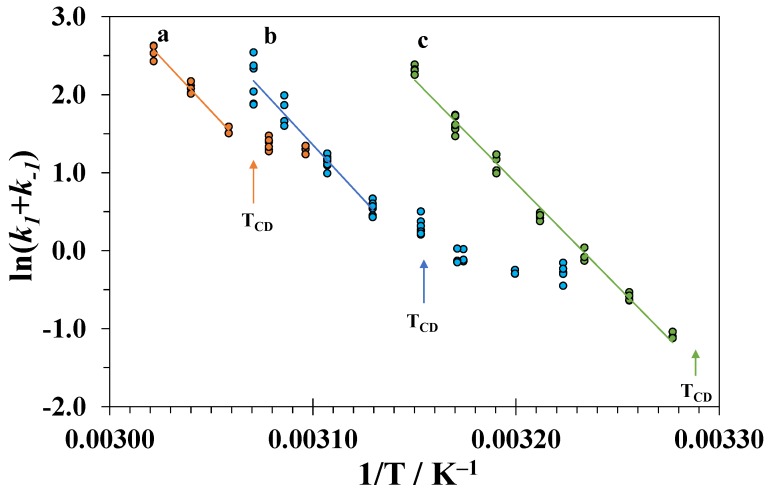
Arrhenius plots for the process of unfolding the tertiary structure of lysozyme in DMSO-water mixtures with different compositions (a − *x*_1_ = 0.25, b − *x*_1_ = 0.30, and c − *x*_1_ = 0.35).

For each composition of the binary mixture and temperature, the measurements were repeated three to four times. The obtained values of the apparent rate constants $k_{1}+k_{-1}$ are plotted in Figure 9 against the inverse temperature 1/*T*.

The linear dependences of $\ln\left(k_{1}+k_{-1}\right)$ on 1/*T* above *T_CD_* were used to determine the activation energies of the tertiary structure unfolding $E_{1}$ and the pre-exponential factor *A*_1_:

(7) $$\ln(k_{1}+k_{-1})\approx \ln k_{1}\left(\mathrm{if}\;T>T_{CD}\right)=\ln A_{1}-\frac{E_{1}}{RT}.$$

The results are presented in Table 4.

The activation energies obtained at different high concentrations of DMSO are close to each other and the value in pure water [62,63]. This means that the difference between the energies of the transition and native state does not depend on the concentration of DMSO. Similar values of the activation energies were also obtained for unfolding of the tertiary structure of lysozyme in water-isopropanol mixtures at pH = 2 [32]. Thus, it is probable that the spatial structure of the transition state and its solvation shell composition remains similar to those in a native state. In contrast, the completely denatured state at a high DMSO content becomes more energetically favorable relative to the native state due to the solvation effects as judged from the low enthalpies of denaturation.

## 4. Conclusions

The study of lysozyme denaturation in the presence of DMSO indicates a non-two-state mechanism of denaturation. The tertiary contacts between amino acid residues start to disrupt before the native structure is completely lost, as suggested from the DSC data. The difference in transition temperatures determined from the near-UV CD and DSC experiments increases with an increasing concentration of DMSO. Moreover, even for denaturation of lysozyme in pure water, some experimental data are inconsistent with the two-state unfolding model.

The addition of DMSO reduces the thermal stability of lysozyme and increases the unfolding rate at a constant temperature. However, due to a decrease in the temperature of denaturation, the unfolding rate at *T_CD_* decreases with a growing concentration of DMSO. A study of kinetics of unfolding in the mixtures with a high content of DMSO showed that even large concentrations of this organic denaturant do not lead to a large change in the activation energy of the tertiary structure unfolding. At the same time, the enthalpy of denaturation of lysozyme in these mixtures is very different from the value in pure water. This supports the hypothesis on the similarity of the structure of the unfolding transition state to the native state.

There are a few other examples of systems in which the activation energy of the protein unfolding does not significantly change with the addition of an organic cosolvent [32,33]. However, many more proteins and cosolvents should be considered before we can conclude how general this tendency is. The data obtained in such experiments can be used to predict the kinetic stability of proteins at different temperatures and at different solvent compositions.

## Figures and Tables

**Table 1:** The temperatures *T_d_* of the lysozyme denaturation peak maximum and the enthalpies *Δ_d_Hcal* of denaturation of lysozyme (per mole of protein) in the mixtures of DMSO with water (mole fraction of DMSO *x*_1_), determined using DSC at a 1 K·min^−1^ heating rate.

*x*_1_	*T_d_* (K)	*Δ_d_H_cal_* (kJ·mol^−1^)
0	351.0	494 ± 10
0.05	347.6	539 ± 9
0.1	344.7	569 ± 10
0.2	337.2	508 ± 9
0.25	331.3	476 ± 9
0.3	326.0	415 ± 12
0.35	319.4	337 ± 18

**Table 2:** The peak maximum temperatures *T_d_* for denaturation of lysozyme in water and in the DMSO–water mixtures (*x*_1_ is a mole fraction of DMSO) at different heating rates in the DSC experiments and the estimates of equilibrium denaturation temperatures *T_d(eq)_*.

*x*_1_	*v* (K·min^−1^)	*T_d_* (K)	*T_d(eq)_*. (K)
0	0.1	350.4	350.3
	0.25	350.4	
	0.5	350.6	
	1.0	351.0	
	2.0	351.4	
0.05	0.5	347.2	347.0
	1.0	347.6	
	2.0	348.1	
0.10	0.5	343.9	343.7
	1.0	344.7	
	2.0	345.2	
0.20	0.5	336.5	336.1
	1.0	337.2	
	2.0	338.1	
0.25	0.5	330.2	329.4
	1.0	331.3	
	2.0	332.9	
0.30	0.5	324.4	323.8
	1.0	326.0	
	2.0	327.2	
0.35	0.5	316.1	312.8
	1.0	319.4	

**Table 3:** The difference between the temperature *T_d_* of the DSC peak maximum (at a 0.5 K·min^−1^ heating rate) and the temperature *T_CD_* corresponding to the maximum of the derivative $\frac{df_{U}}{dT}$ for DMSO–water mixtures with different mole fractions of DMSO *x*_1_.

*x*_1_	*T_d_* − *T_CD_* (K)
0	0.6
0.1	1.7
0.2	3.6
0.25	4.4
0.3	7.3
0.35	11.7

**Table 4:** The activation energies and the pre-exponential factors for the process of disruption of the tertiary structure of lysozyme in the DMSO–water mixtures with a different mole fraction of DMSO *x*_1_.

*x*_1_	*E*_1_ (kJ·mol^−1^)	ln(*A*_1_/min^−1^)
0.25	231 ± 20	86.6 ± 4
0.30	233 ± 18	88.5 ± 4
0.35	222 ± 15	85.7 ± 2

## Supplementary Materials

The following are available online at https://www.mdpi.com/2218-273X/9/10/547/s1. Figures of temperature dependences of molar ellipticity of hen egg-white lysozyme at 290 nm in DMSO-water mixtures.
