# Supplementary material for: The Effect of Dimethyl Sulfoxide on the Lysozyme Unfolding Kinetics, Thermodynamics, and Mechanism
Source: Biomolecules. 2019 Sep 29;9(10):547. doi: 10.3390/biom9100547 (PMC6843525; doi:10.3390/biom9100547)
Supplement: Supplementary file 1 [file biomolecules-09-00547-s001.pdf]

Temperature dependences of molar ellipticity of hen egg-white lysozyme at 290 nm in DMSO-water mixtures.

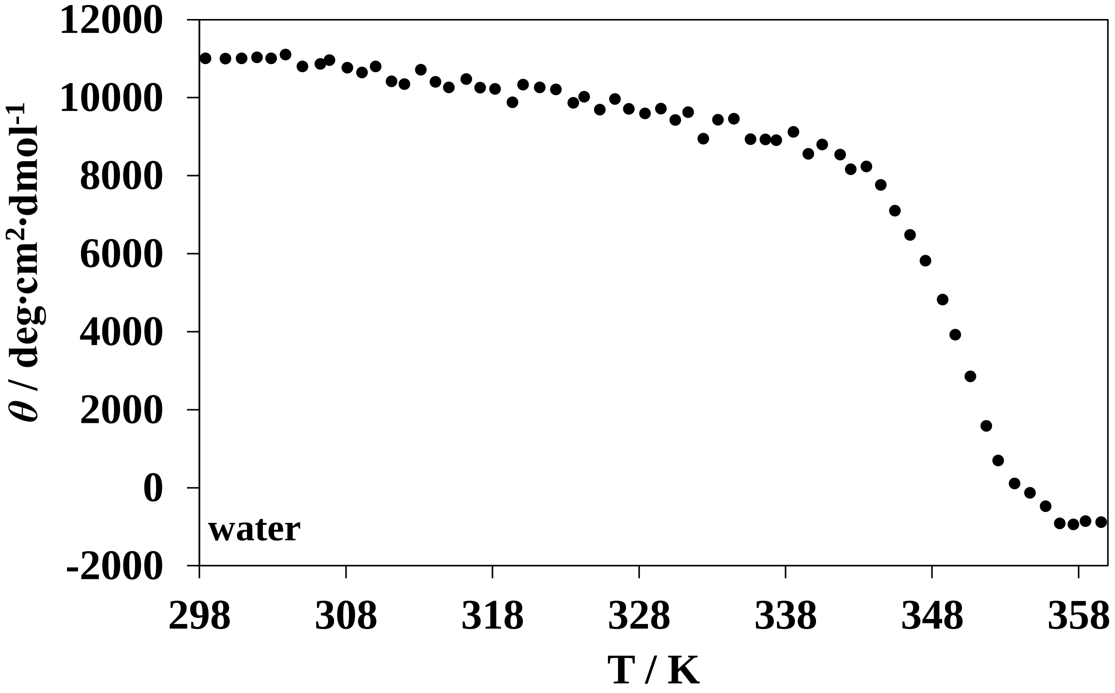

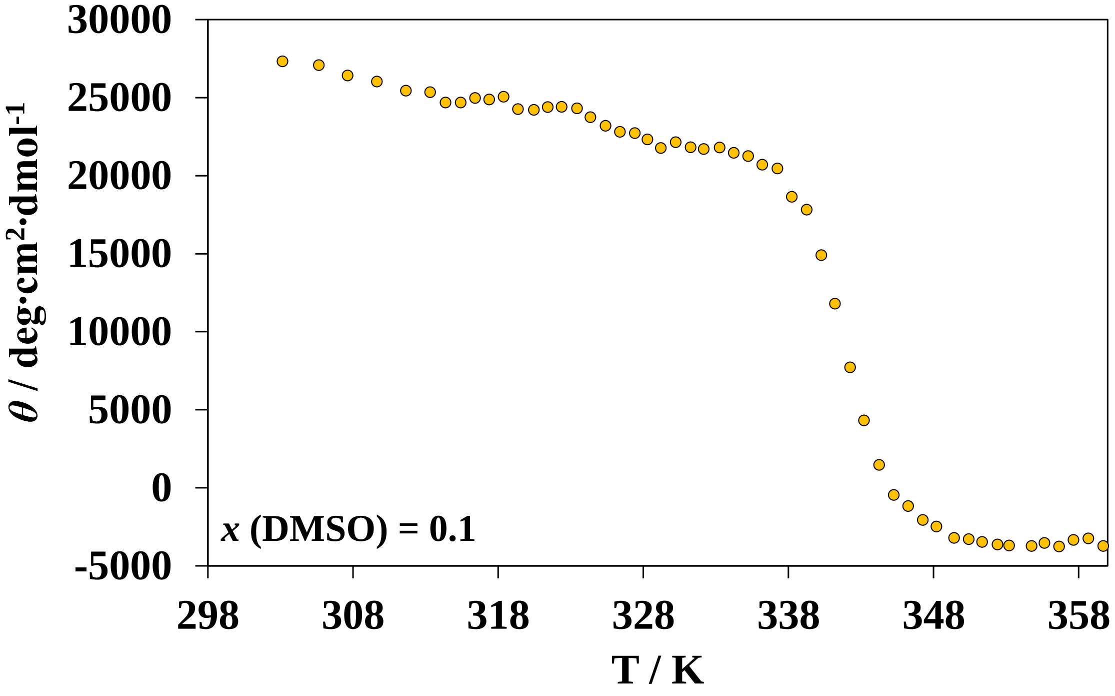

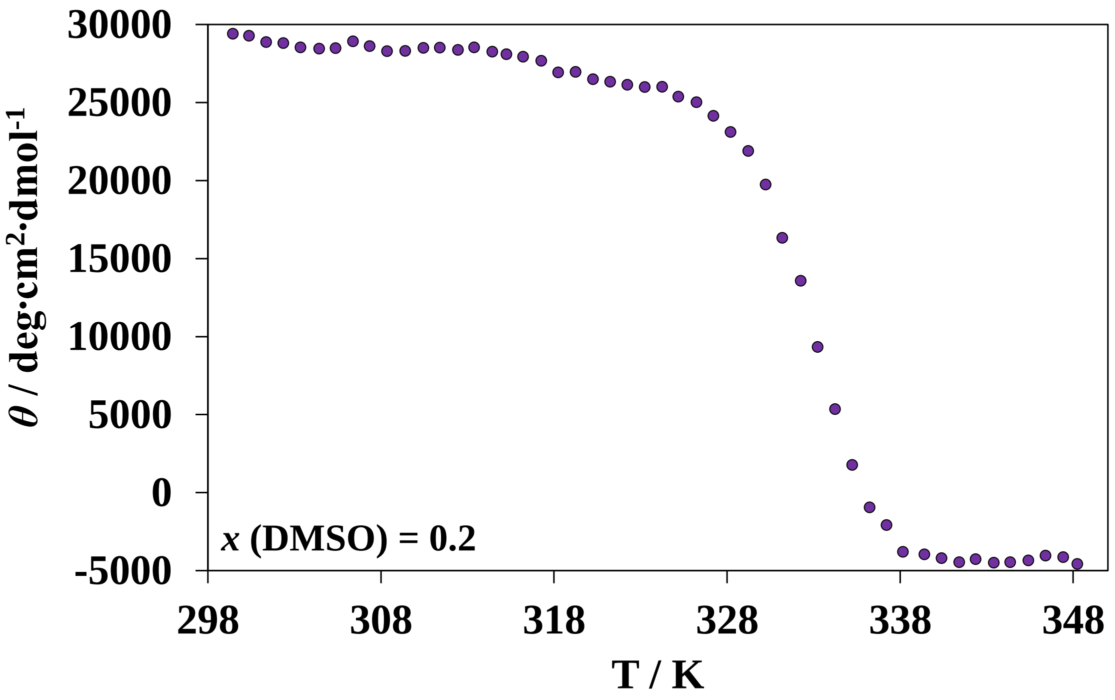

30000
25000
20000
15000
10000
5000
0
-5000
θ / deg·cm2·dmol-1
x (DMSO) = 0.2
298
308
318
328
338
348
T / K

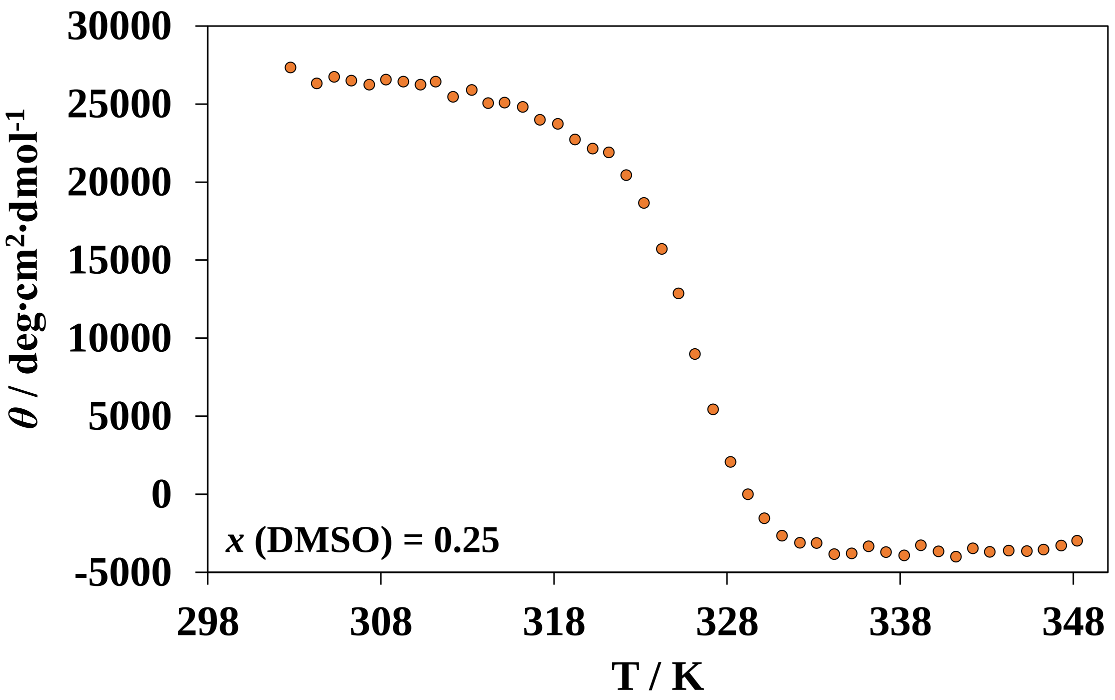

30000
25000
20000
15000
10000
5000
0
-5000
θ / deg·cm2·dmol-1
x (DMSO) = 0.25
298
308
318
328
338
348
T / K

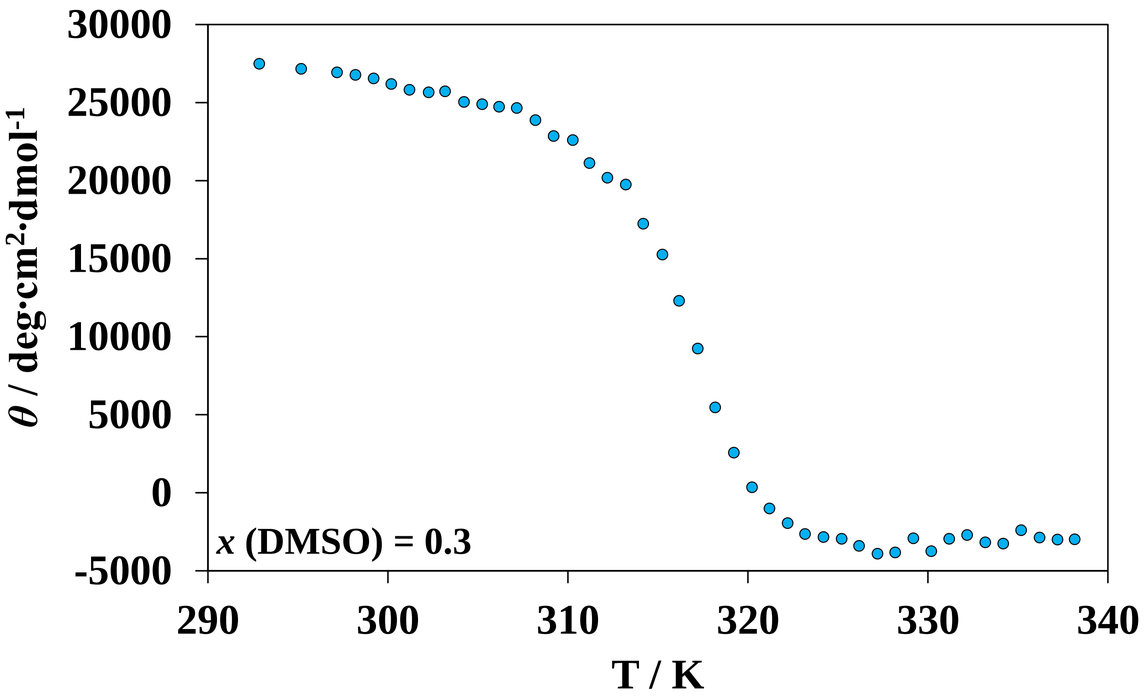

30000
25000
20000
15000
10000
5000
0
-5000
θ / deg·cm²·dmol⁻¹
x (DMSO) = 0.3
290
300
310
320
330
340
T / K

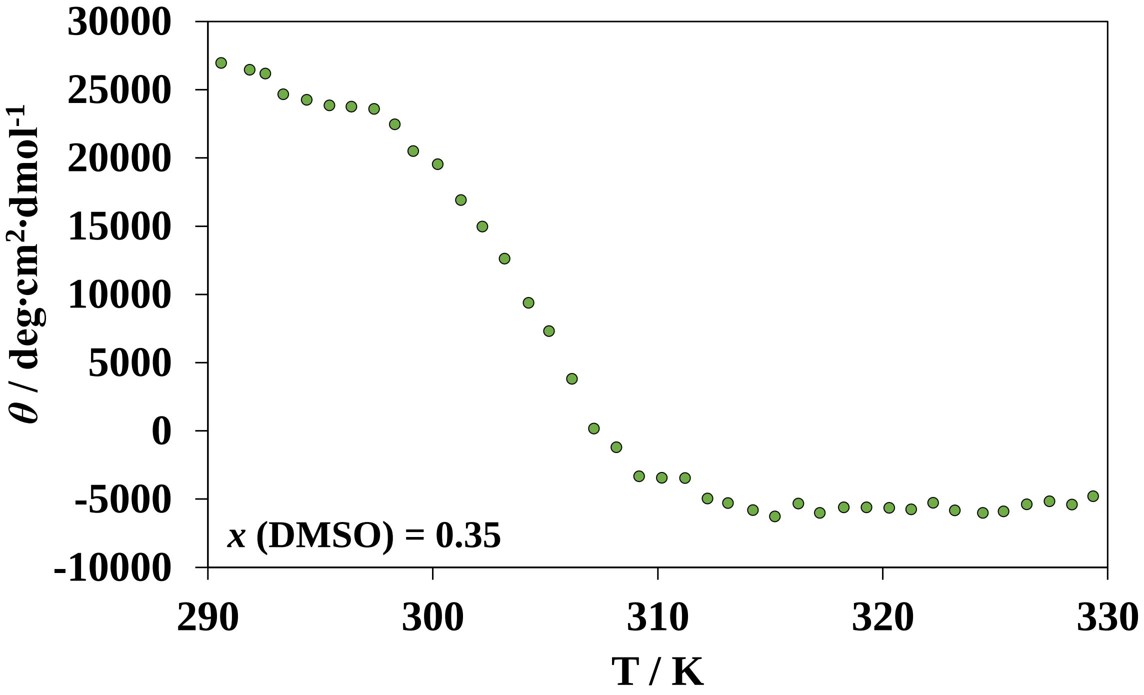

30000
25000
20000
15000
10000
5000
0
-5000
-10000
θ / deg·cm²·dmol⁻¹
x (DMSO) = 0.35
290
300
310
320
330
T / K
